# Supplementary material for: The Evolution and Characterization of the RNA Interference Pathways in Lophotrochozoa
Source: Genome Biol Evol. 2024 May 7;16(5):evae098. doi: 10.1093/gbe/evae098 (PMC11114477; doi:10.1093/gbe/evae098)
Supplement: evae098_Supplementary_Data [file evae098_supplementary_data.zip › Supplementary_materials.zip!/selected_miRNAs/NoGe_selected_miRNAs/mirdeep_pdf_files/NMRB01005527.1_Notospermus_geniculatus_strain_Ushimado_scaffold5527,_whole_genome_shotgun_sequence_47697.pdf]

Provisional ID : NMRB01005527.1_Notospermus_geniculatus_strain_Ushimado_scaffold5527,_whole_genome_shotgun_sequence_47697
Score total : 110.4
Score for star read(s) : 3.9
Score for read counts : 104.6
Score for mfe : 2.4
Score for randfold : 0
Score for cons. seed : -0.6
Total read count : 217
Mature read count : 156
Loop read count : 0
Star read count : 61

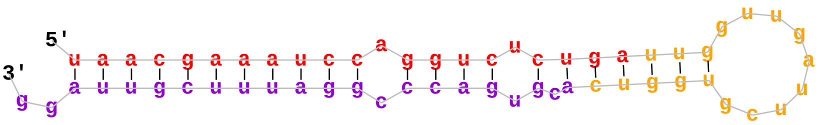

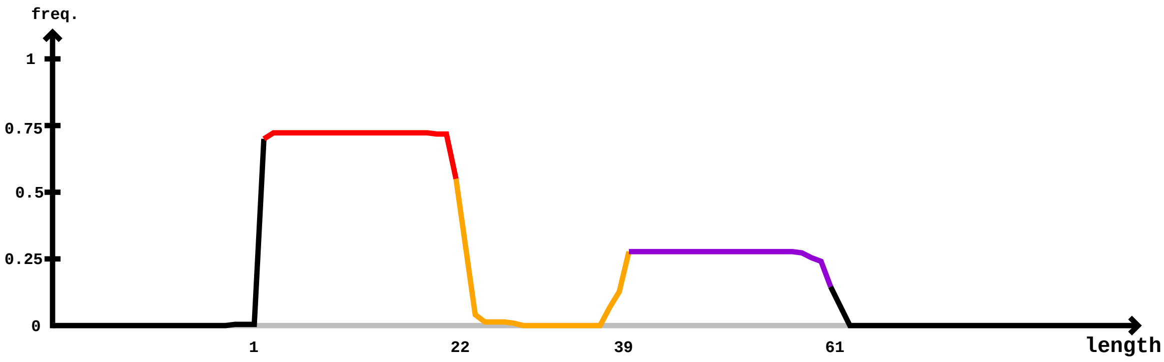


```
                           Mature                                   Star
5'- augcugaccuguaccugucc uaacgaaauccaggucucuga uugguugauucgugguc acgugacccggauuucguuagg ucagguacagguacgucgacaagauggcggc -3'  obs
```

```
5'- augcugaccuguaccuguccuaacgaaauccaggucucugauugguugauucguggucacgugacccggauuucguuaggucagguacagguacgucgacaagauggcggc -3'   obs
    augcugaccuguaccuguccuaacgaaauccaggucucugauugguugauucguggucacgugacccggauuucguuaggucagguacagguacgucgacaagauggcggc         exp
    ..(((((((((((((((.(((((((((((((.((((.(((((((.........)))))).).)))).))))))))))))).))))))))))).((((.....)))).))))    reads   mm   sample
    .....................aacgaaauccaggucucuga......................................................................    1       0    Fm3
    ........................................................ucacgugacccggauuucguu..................................    4       0    Fm3
    ........................................................ucacgugacccggauuucguuaU................................    3       1    Fm3
    .........................................................cacgugacccggauuucguuagU...............................    2       1    Fm3

    .................uccuaacgaaaGccaggucucugauuggu.................................................................    1       1    Mm3
    ....................uaacgaaauccaggucucug.......................................................................    24      0    Mm3
    ....................uaacgaaauccGggucucug.......................................................................    1       1    Mm3
    ....................uaacgaaauccGggucucuga......................................................................    22      1    Mm3
    ....................uaacgaaauccaggucucugG......................................................................    2       1    Mm3
    ....................uaacgaaaucAaggucucuga......................................................................    1       1    Mm3
    ....................uaacgaaauccaggucucuga......................................................................    36      0    Mm3
    ....................uaacgaaGuccaggucucuga......................................................................    1       1    Mm3
    ....................uaacgaaauccaggucucugaA.....................................................................    4       1    Mm3
    ....................uaacgaaauccGggucucugau.....................................................................    4       1    Mm3
    ....................uaacgaaauccaggucucugau.....................................................................    13      0    Mm3
    ....................uaacgaaauccaggucCcugau.....................................................................    1       1    Mm3
    ....................uaacgaaGuccaggucucugau.....................................................................    1       1    Mm3
    ....................uaacgaaauccaggucuUugau.....................................................................    1       1    Mm3
    ....................uaacgaaauccaggucAcugau.....................................................................    1       1    Mm3
    ....................uaacgaaauccaggucucugaAu....................................................................    2       1    Mm3
    .....................aacgaaauccaggucucugauu....................................................................    1       0    Mm3
    ........................................................ucacgugacccggauuucguuag................................    2       0    Mm3
    ........................................................ucacgugacccggauuucguuagU...............................    1       1    Mm3
    .........................................................cacgugacccggauuucguuag................................    4       0    Mm3
    .........................................................cacgugacccggauuucAuuag................................    1       1    Mm3
    .........................................................cacgugacccggauuucguuagU...............................    2       1    Mm3
    ..........................................................acgugacccggauuucguuagg...............................    5       0    Mm3
    ..........................................................acgugacccggauuucgAuagg...............................    1       1    Mm3
    ..........................................................acgugacccggauuucguuagU...............................    5       1    Mm3
    ..........................................................acgugacccggauuucguuagA...............................    3       1    Mm3
    ..........................................................acgugacccggauuucguuagg...............................    4       0    Mm3

    ....................uaacgaaauccaggucucugau.....................................................................    2       0    Mm1
```

Mature Star

augcugaccuguaccuguccuaacgaaauccaggucucugauugguugauucgugguacgugacccggauuucguuaggucagguacagguacgucgacaagauggcggc			
....................uGacgaaauccaggucucugau...	5	1	Mm1
....................uaacgaaauccaggucuc...	1	0	Mm2
....................uaacgaaauccaggucucug...	12	0	Mm2
....................uaacgaaGuccaggucucuga..	2	1	Mm2
....................uaacgaaauccGggucucuga..	5	1	Mm2
....................uaacgaaauccaggucucuga..	5	0	Mm2
....................uaacgaaauccaggucucugau...	1	0	Mm2
....................uaacgaaauccaggucucugaA...	2	1	Mm2
....................uaacgaaauccGggucucugau...	1	1	Mm2
....................uaacgaaauccGggucucugauu..	1	1	Mm2
....................uaacgaaaGccaggucucugauugguu..	1	1	Mm2
....................uaacgaaauccGggucucugauugguu..	1	1	Mm2
.....................aacgaaauccGggucucugau...	1	1	Mm2
.....................aacgaaauccaggucucugauu..	2	0	Mm2
....ucacgugacccggauuucguuU.................................	1	1	Mm2
....ucacgugacccggauuucguua.................................	2	0	Mm2
....ucacgugacccggauuucguAag................................	1	1	Mm2
....ucacgCgacccggauuucguuag................................	1	1	Mm2
....cacgugacccggauuucgu...................................	1	0	Mm2
....cacgCgacccggauuucguuag................................	1	1	Mm2
....cacgugacccggauuucguuagA...............................	1	1	Mm2
....cacgugacccggauuuUguuagg...............................	1	1	Mm2
....acgCgacccggauuucguuag................................	2	1	Mm2
....acgugacccggauuucguuag................................	1	0	Mm2
....acgugacccggauuucguuagg...............................	5	0	Mm2
....acgCgacccggauuucguuagg...............................	6	1	Mm2
....acgugacccggauuucguuGggu..............................	1	1	Mm2
